# LCK and CD3E Orchestrate the Tumor Microenvironment and Promote Immunotherapy Response and Survival of Muscle-Invasive Bladder Cancer Patients

**DOI:** 10.3389/fcell.2021.748280

**Published:** 2021-12-24

**Authors:** Xiaonan Zheng, Xinyang Liao, Ling Nie, Tianhai Lin, Hang Xu, Lu Yang, Bairong Shen, Shi Qiu, Jianzhong Ai, Qiang Wei

**Affiliations:** ^1^ Department of Urology, Institute of Urology, West China Hospital, Sichuan University, Chengdu, China; ^2^ Institute of Systems Genetics, West China Hospital, Sichuan University, Chengdu, China; ^3^ Department of Pathology, West China Hospital, Sichuan University, Chengdu, China

**Keywords:** muscle-invasive bladder cancer, LCK, CD3e, tumor microenvironment, immunotherapy

## Abstract

**Background:** Studies have demonstrated the significance of multiple biomarkers for bladder cancer. Here, we attempt to present biomarkers potentially predictive of the prognosis and immunotherapy response of muscle-invasive bladder cancer (MIBC).

**Method:** Immune and stromal scores were calculated for MIBC patients from The Cancer Genome Atlas (TCGA). Core differential expression genes (DEGs) with prognostic value were identified and validated using an independent dataset GSE31684. The clinical implications of prognostic genes and the inter-gene correlation were presented. The distribution of tumor-infiltrating immune cells (TICs), the correlation with tumor mutation burden (TMB), and the expression of eight immune checkpoint–relevant genes and CD39 were accordingly compared. Two bladder cancer cohorts (GSE176307 and IMvigor210) receiving immunotherapy were recruited to validate the prognostic value of LCK and CD3E for immunotherapy.

**Results:** 361 MIBC samples from TCGA revealed a worse overall survival for higher stromal infiltration (*p* = 0.009) but a better overall survival for higher immune infiltration (*p* = 0.042). CD3E and LCK were independently validated by TCGA and GSE31684 to be prognostic for MIBC. CD3E was the most correlative gene of LCK, with a coefficient of r = 0.86 (*p* < 0.001). CD8^+^ T cells and macrophage M1 are more abundant in favor of a higher expression of CD3E and LCK in MIBC and across pan-cancers. Immune checkpoints like CTLA4, CD274 (PD-1), and PDCD1 (PD-L1) were highly expressed in high-CD3E and high-LCK groups for MIBC and also for pan-cancers, except for thymoma. LCK and CD3E had a moderate positive correlation with CD39 expression. Importantly, high-LCK and high-CD3E groups had a higher percentage of responders than the low-expression groups both in GSE176307 (LCK: 22.73*vs.* 13.64%, CD3E: 22.00 *vs.* 13.16%) and IMvigor210 cohorts (LCK: 28.19 *vs.* 17.45%, CD3E: 25.50 *vs.* 20.13%).

**Conclusion:** CD3E and LCK were potential biomarkers of MIBC. CD3E and LCK were positively correlated with several regular immunotherapy biomarkers, which is supported by real-world outcomes from two immunotherapy cohorts.

## Introduction

As one of the most common malignant solid tumors, bladder cancer (BC) causes 573,278 incidents and 212,536 deaths in 2020 (Sung et al., 2021). Muscle-invasive bladder cancer (MIBC), the advanced stage of BC, makes up 20–30% of BC at the initial diagnosis, and the five-year overall survival is maintained less than 50% (Lenis et al., 2020). Although radical cystectomy still remains a common approach, immunotherapy has rapidly progressed, with five immune checkpoint inhibitors approved to treat advanced BC. However, it is noticeable that the response rate to those immunotherapy drugs reaches only 20–40%, which greatly restricts the clinical management of MIBC (Doroshow et al., 2021).

Studies have demonstrated the positive role of multiple biomarkers such as tumor mutation burden (TMB) (Goodman et al., 2017), the abundance of tumor-infiltrating immune cells (TICs) (Petitprez et al., 2020), and the expression level of PD-L1 (Jia et al., 2018) and CD39 (also known as ENTPD1) (Allard et al., 2017; Moesta et al., 2020) in predicting the response rate to immune checkpoint inhibitors (ICIs). However, the limited accuracy of those biomarkers should still be noted. In recent years, immune cells have been recognized as a key component of the tumor microenvironment. Immune cells are essentially involved in tumorigenesis and tumor progression and thus influence the survival outcomes (Fu et al., 2018; Hinshaw and Shevde, 2019; Jiang et al., 2019). Based on this concept, it appears reasonable to identify prognostic biomarkers for MIBC by predicting the level of TICs (Yoshihara et al., 2013). With these immune-based prognostic genes, we can further estimate their correlation with immune checkpoint genes, tumor mutation burden, TICs, and certain predictors such as CD39, which may hopefully provide us new insights into the precise immunotherapy of MIBC. Therefore, this study aims to identify new immune-based prognostic genes of MIBC and validate them in clinical cohorts receiving immunotherapy.

## Methods

### Data Collection and Immune and Stromal Score Calculation

Gene expression data of bladder cancer with clinical variables were obtained from The Cancer Genome Atlas (TCGA) database, and patients with MIBC (T2 to T4) were selected for subsequent analysis. Another independent dataset was downloaded from Gene Expression Omnibus (GEO) for external validation. The ESTIMATE algorithm was used to calculate scores to predict the level of immune and stromal cell infiltration for each patient (Yoshihara et al., 2013). The R package “ESTIMATE” has been widely utilized in cancer-related studies (Liu et al., 2021a; Liu et al., 2021b; Liu et al., 2021c). The optimal cutoff values of immune and stromal scores were determined with maximally selected log-rank using the R package “survminer” (Li et al., 2020), and patients were then divided into immune-high/-low and stromal-high/-low groups. Kaplan–Meier curves were utilized to evaluate the association of immune and stromal scores with survival outcomes.

### Identification of Prognostic Genes

Differential expression genes (DEGs) between immune-high versus immune-low and stromal-high versus stromal-low subgroups were identified using the “limma” R package with a setting of |fold change| > 2 and a *p* value < 0.05, visualized with a heatmap and intersected with a Venn plot. Enrichment analyses of gene ontology (GO) and the Kyoto Encyclopedia of Genes and Genomes (KEGG) pathway were performed to reveal the biological process, cellular component, molecular function, and molecular pathways that the intersected DEGs were associated with.

The protein–protein interaction (PPI) network of the DEGs was constructed with the STRING database (https://string-db.org/), with an interaction confidence of 0.99, and the core modules of the network were identified, defined as a collection of genes with no less than three nodes within the network. The prognostic value of the genes present in the core modules of the PPI network was evaluated with Kaplan–Meier curves. Then an independent GEO dataset GSE31684 was used for the external validation of the prognostic genes. A *p* value < 0.05 indicates that the correlation is significant.

### Clinical and Immune Implications of the Prognostic Genes

The first 100 most correlative genes of each prognostic gene were identified and then intersected. Subsequent enrichment analyses and network construction were performed. The expression of the prognostic genes was compared between tumor and normal samples with data downloaded from the UALCAN cancer database (Chandrashekar et al., 2017) and the Human Protein Atlas (Uhlén et al., 2015). The connection between the prognostic genes and clinical variables was presented in a Sanguini diagram using the R package “ggalluval.” The percentage abundance of TICs was predicted and displayed using the R package “pheatmap,” and the CIBERSORT algorithm was utilized to compare the distribution of TICs according to the expression level of prognostic genes with the R package “ggplot.” We further evaluated the expression of eight immune checkpoint–relevant genes to reveal a potential role of the prognostic genes in immunotherapy. Correlation between TICs, immune checkpoint–relevant genes expression levels, and TMB with LCK and CD3E was further performed for pan-cancers, including 32 kinds of tumors. Another two independent bladder cancer cohorts receiving anti-PD1/PD-L1 inhibitor immunotherapy (GSE176307 (Rose et al., 2021) and IMvigor210 (Mariathasan et al., 2018)) were recruited to validate the predictive value of LCK and CD3E for immune response (responder: partial response [PR] or complete response [CR]; non-responder: stable disease [SD]; or progressed disease [PD]). The R package “Maxstat,” “Survminer,” “Survival,” and “ggplot2” were used to assess the prognostic significance of LCK and CD3E. The correlation of LCK and CD3E with immune phenotypes (inflamed, excluded, and desert) in the IMvigor210 cohort was also analyzed.

## Result

### CD3E and LCK Are the Prognostic Genes of MIBC

**FIGURE 1:**
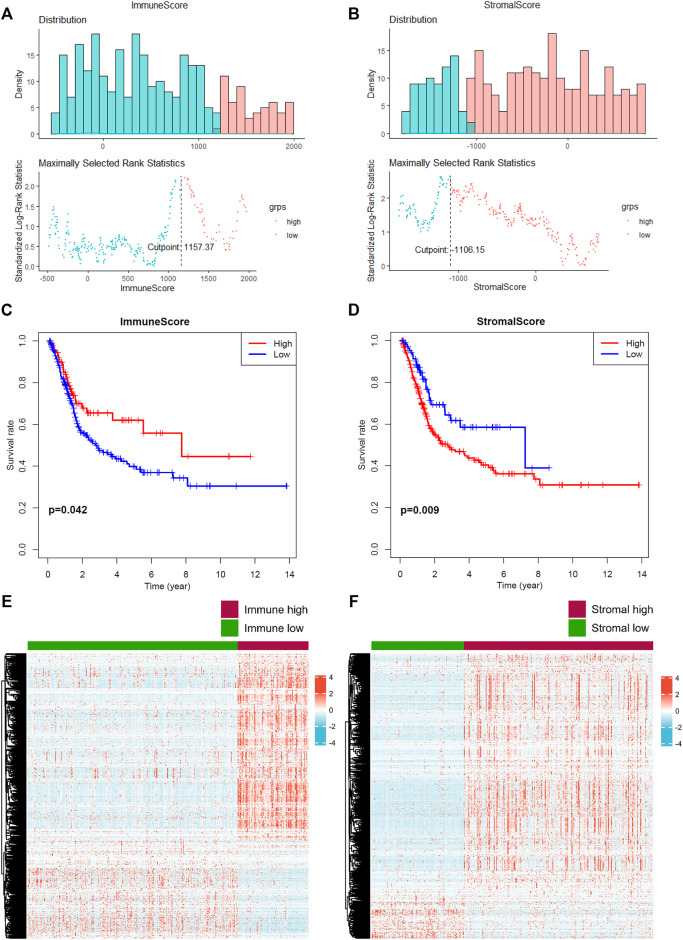
Prediction of the level of tumor-infiltrating immune cells and stromal cells. **(A–B)** The optimal cutoff value of the immune score and stromal score was calculated. **(C–D)** Prognostic value of the immune score and stromal score. **(E–F)** Identification of differential expressed genes according to the immune and stromal scores. A *p* value < 0.05 indicates statistical significance.

A total of 361 and 78 MIBC patients were identified from TCGA and GSE31684 (Table 1), respectively. The optimal cutoff values were 1157.37 for the immune score and -1106.15 for the stromal score (Figures 1A, B). In total, 92 patients from TCGA were assigned to the immune-high group and 269 to the immune-low group, while 253 patients were classified as stromal-high and 108 stromal-low. Higher immune infiltration was associated with improved overall survival (*p* = 0.042), while increased stromal infiltration was associated with worse overall survival (*p* = 0.009) (Figures 1C, D).

**TABLE 1:** Characteristics of included patients from TCGA and GSE31684 datasets.

	TCGA MIBC	GEO GSE31684
Age (years)		
<65	129 (35.73%)	24 (30.77%)
≥65	232 (64.27%)	54 (69.23%)
Sex		
Female	95 (26.32%)	21 (26.92%)
Male	266 (73.68%)	57 (73.08%)
T stage		
T2	114 (31.58%)	17 (21.79%)
T3	190 (52.63%)	42 (53.85%)
T4	57 (15.79%)	19 (24.36%)
Mean follow-up (days)	785.07	1209.45

**FIGURE 2:**
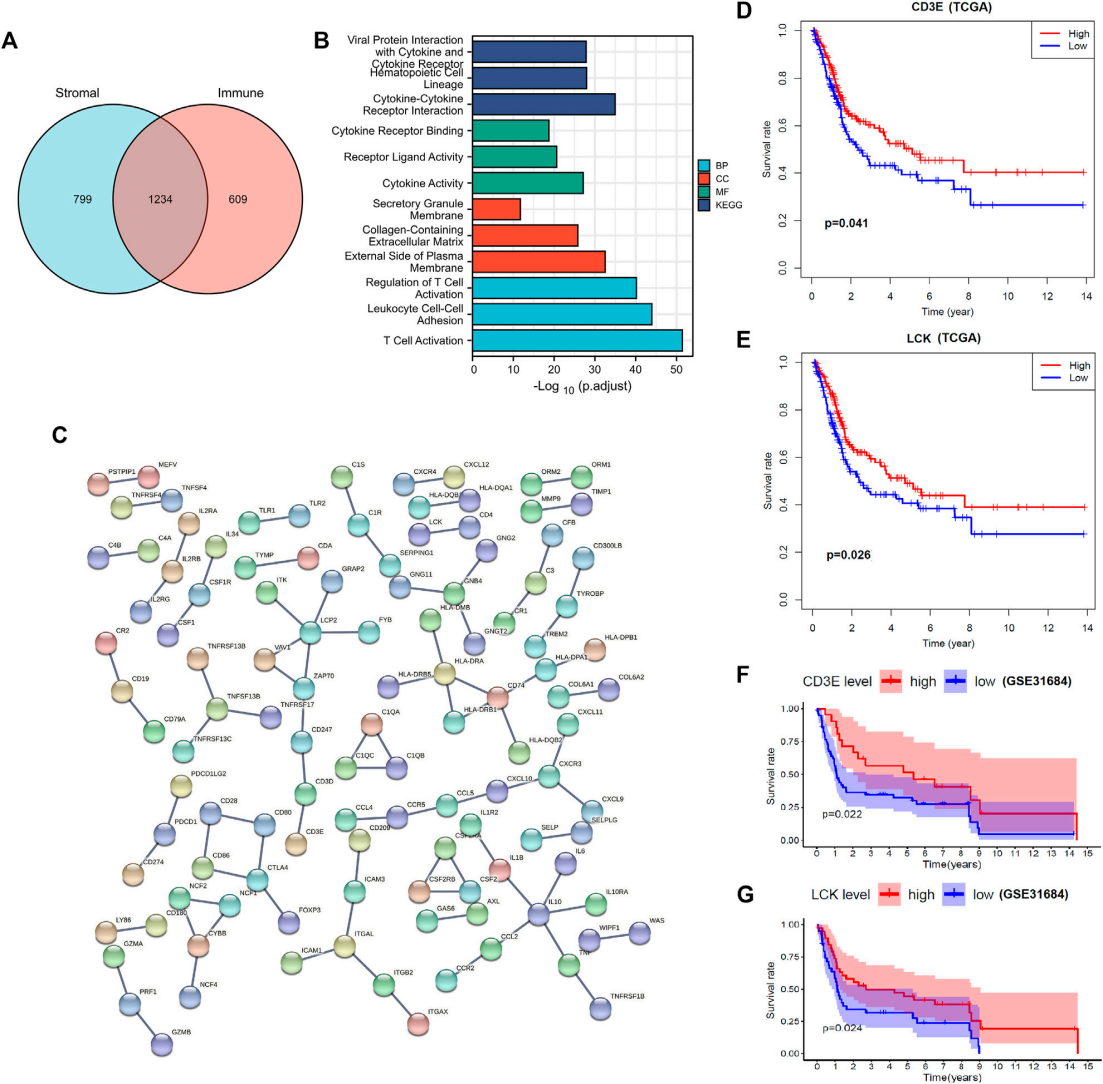
Identification and validation of prognostic genes of muscle-invasive bladder cancer. **(A)** Intersection of differential expressed genes. **(B–C)** Enrichment analyses and the protein–protein network construction of the intersected differential expressed genes. **(D–E)** Partial presentation of the prognostic genes with the core module of the protein–protein network found through TCGA samples. **(F–G)** External validation with the GSE31684 dataset identifies that LCK and CD3E were the prognostic genes of muscle-invasive bladder cancer. A *p* value < 0.05 indicates statistical significance.

In total, 2033 DEGs were identified in stromal-high/low groups and 1843 DEGs in the immune-high/low groups (Figures 1E, F). Then 1234 DEGs were eventually intersected (Figure 2A), and the enrichment analyses indicated a role in cytokine–cytokine receptor interaction and T-cell activation (Figure 2B). Network construction identified a core module of 173 genes (Figure 2C). Survival curves demonstrated that CD3E (TCGA: *p* = 0.041, GEO: *p* = 0.022) and LCK (TCGA: *p* = 0.026, GEO: *p* = 0.024) were the prognostic genes of MIBC after external validation with GSE31684 (Figures 2D–G).

### CD3E Is the Most Correlative Gene of LCK in MBC

**FIGURE 3:**
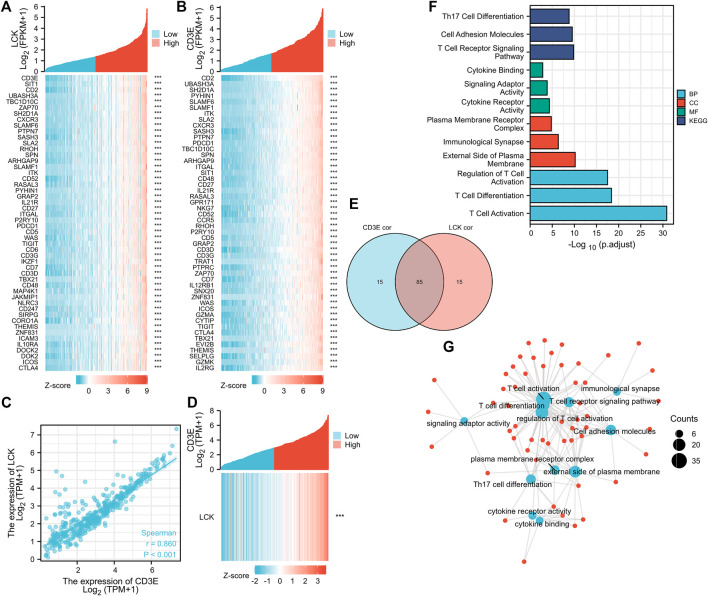
Correlation between LCK and CD3E. **(A–B)** co-expressed genes of LCK and CD3E. **(C–D)** Spearman correlation and co-expression analysis between LCK and CD3E. **(E)** Intersection of the first 100 genes co-expressed with LCK and CD3E. **(F–G)** Enrichment analyses and the network pathway of the 85 intersected genes.

The top 100 genes that co-express with LCK and CD3E are partially presented in Figures 3A, B. Interestingly, CD3E was the most correlative gene of LCK, with a Spearman coefficient r = 0.86 (*p* < 0.001) (Figures 3C, D). The intersection of the top 100 co-expressed genes of LCK and CD3E were found to be associated with T-cell activation and differentiation and the T-cell receptor signaling pathway (Figures 3E–G).

### Correlation of LCK/CD3E with Clinical Characteristics of MIBC

**FIGURE 4:**
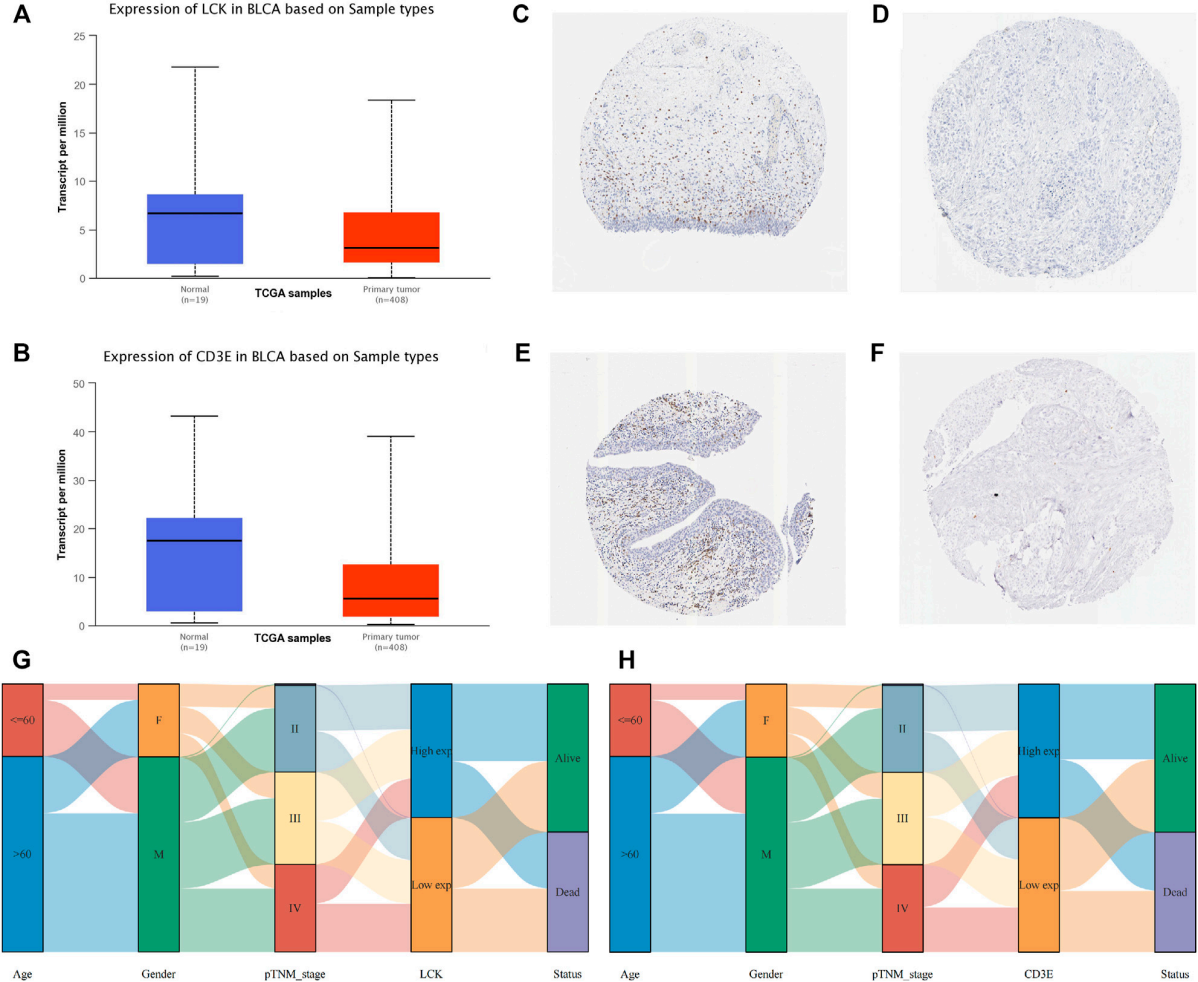
Clinical implication of LCK and CD3E. **(A)** Comparison of the expression value of LCK between normal tissue and bladder cancer tissue from TCGA; immunohistochemical staining of LCK in normal tissue **(C)**and bladder tumor tissue **(D)**. **(B)** Comparison of the expression value of CD3E between normal tissue and bladder cancer tissue from TCGA. Immunohistochemical staining of LCK in normal tissue **(E)** and bladder tumor tissue **(F)**. **(G–H)** Correlation of LCK and CD3E with clinical information of muscle-invasive bladder cancer.

The expression of LCK and CD3E was found to be much lower in tumor samples than in normal samples, with a median expression value (transcript per million) of 6.685 vs 3.116 for LCK (Figure 4A) and 17.484 vs 5.58 for CD3E (Figure 4B). Immunohistochemical staining showed a consistent expression trend of LCK (Figures 4C, D) and CD3E (Figures 4E, F) between normal tissue and bladder cancer samples. The association between clinical variables, including age, gender, and pathological stage, with the expression of LCK and CD3E, was also displayed, from which we could observe that there was a tendency of the distribution of high/low LCK/CD3E across different pathologic stages of MIBC (Figures 4G, H). Supplementary Figure S1 more quantitatively shows that MIBC patients in earlier stages (stages II and III vs stage IV) had a slightly higher percentage of high-LCK and high-CD3E expression (high-LCK: 53.11 *vs.* 44.19%; high-CD3E: 51.55*vs.*vs 48.06%; Supplementary Figure S1A). Further subgroup analyses consistently indicated that higher expressions of LCK and CD3E were found in MIBC patients diagnosed with earlier T stage (T2&T3 *vs.* T4: high-LCK: 50.16*vs.*vs 48.28%; high-CD3E: 50.32 vs 46.55%; Supplementary Figure S1B), and in particular earlier N stage (N0 *vs.* N1 and N2 *vs.* N3: high-LCK: 52.04 *vs.* 46.22*vs.*vs 28.57%; high-CD3E: 51.58 vs 49.58% *vs.* 28.57%; Supplementary Figure S1C) and earlier M stage (M0 *vs.* M1: high-LCK: 45.76 *vs.* 0%; high-CD3E: 42.94 *vs.* 12.50%; Supplementary Figure S1D).

### Correlation of LCK/CD3E with Tumor-Infiltrating Lymphocytes, Immune Checkpoint, Tumor Mutation Burden, and ENTPD1 (CD39)

**FIGURE 5:**
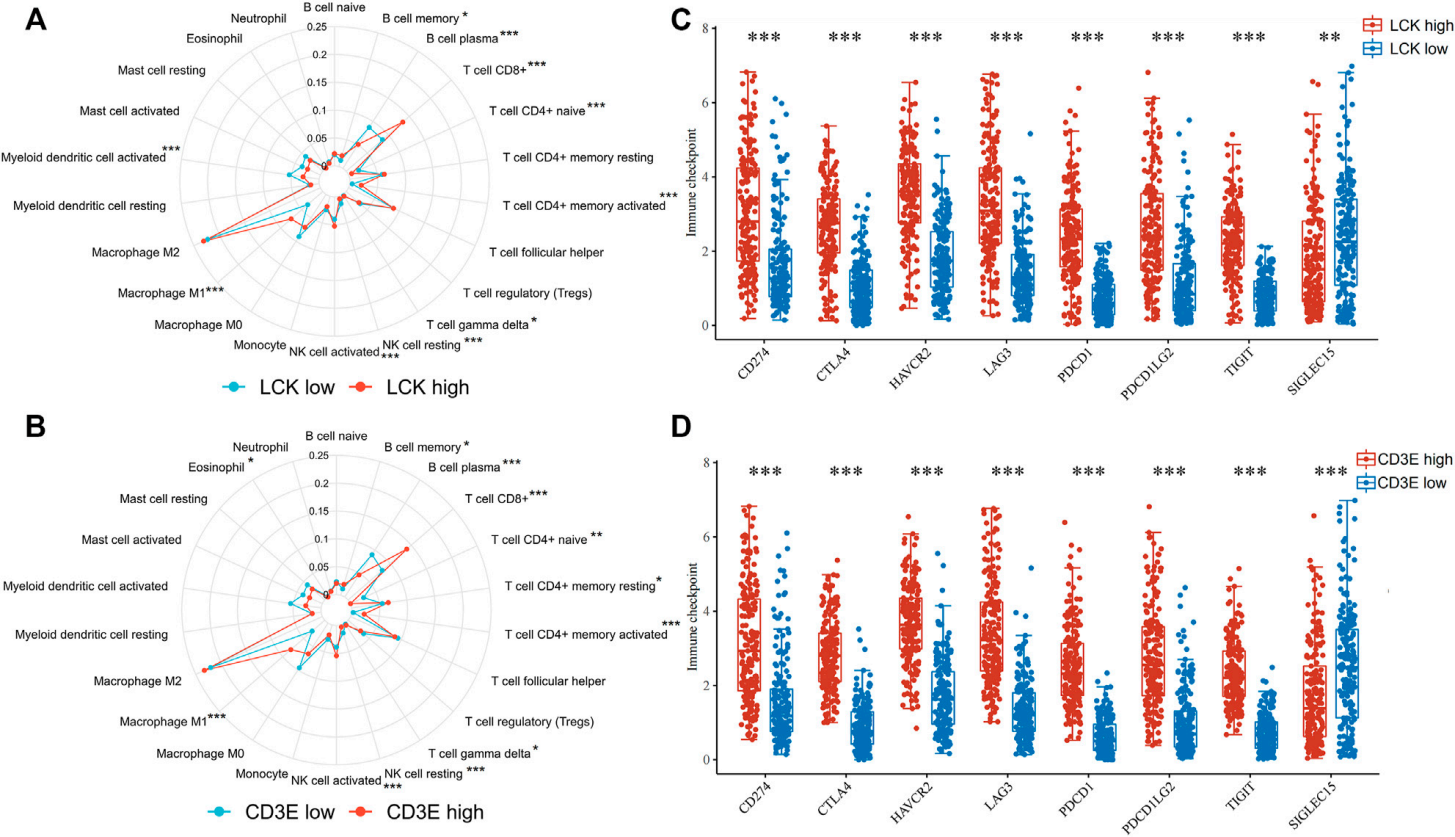
Association of LCK and CD3E with the distribution of tumor-infiltrating immune cells and with the expression of immune checkpoint genes in muscle-invasive bladder cancer. **(A–B)** Distribution of tumor-infiltrating immune cells based on the expression level of LCK and CD3E. **(C–D)** Expression level of immune checkpoint genes based on the expression level of LCK and CD3E. * *p* < 0.05, ** *p* < 0.01, *** *p* < 0.001.

**FIGURE 6:**
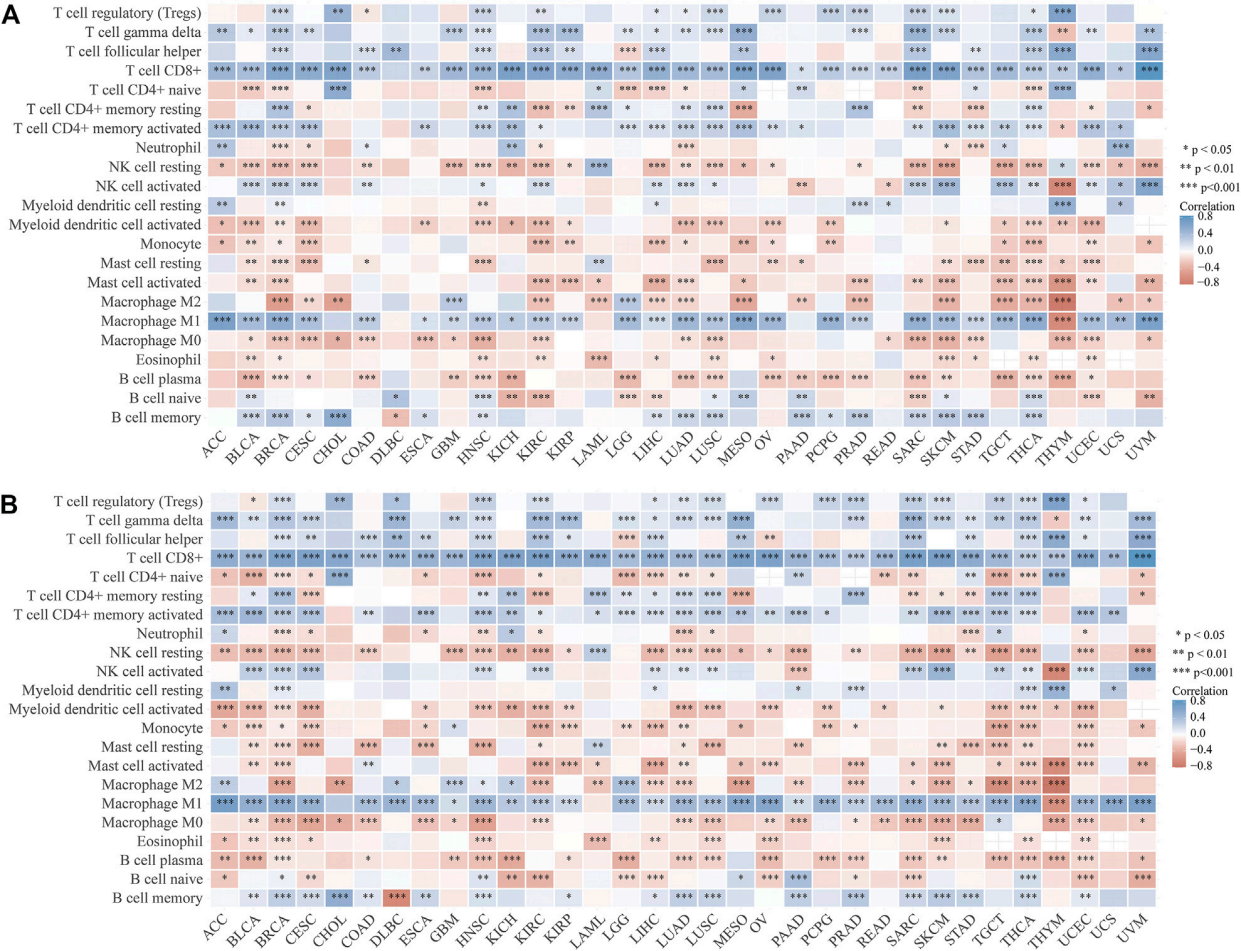
Correlation between tumor-infiltrating immune cells with the expression of LCK **(A)** and CD3E **(B)** across pan-cancers.

The percentage abundance of TICs in MIBC patients is shown in Supplementary Figure S2 and Supplementary Table S1, from which we have a general view of the percentage of different TICs in MIBC samples. The CIBERSORT algorithm revealed the significantly different distribution of TICs between LCK-high/low and CD3E-high/low subgroups; for instance, the infiltration of memory B cells, CD8^+^ T cells, CD4^+^memory activated, and macrophage M1 was higher in the LCK-high and CD3E-high subgroups (Figures 5A, B). Further analysis indicated a higher expression of several key immune checkpoint–relevant genes such as PD-1, PD-L1, PD-L2, and CTLA4 in LCK-high and CD3E-high subgroups (Figures 5C, D). Moreover, this positive correlation between memory B cell, CD8^+^ T cell, CD4^+^ memory activated, and macrophage M1 with LCK and CD3E was also consistent across different cancer types (Figures 6A,B).

**FIGURE 7:**
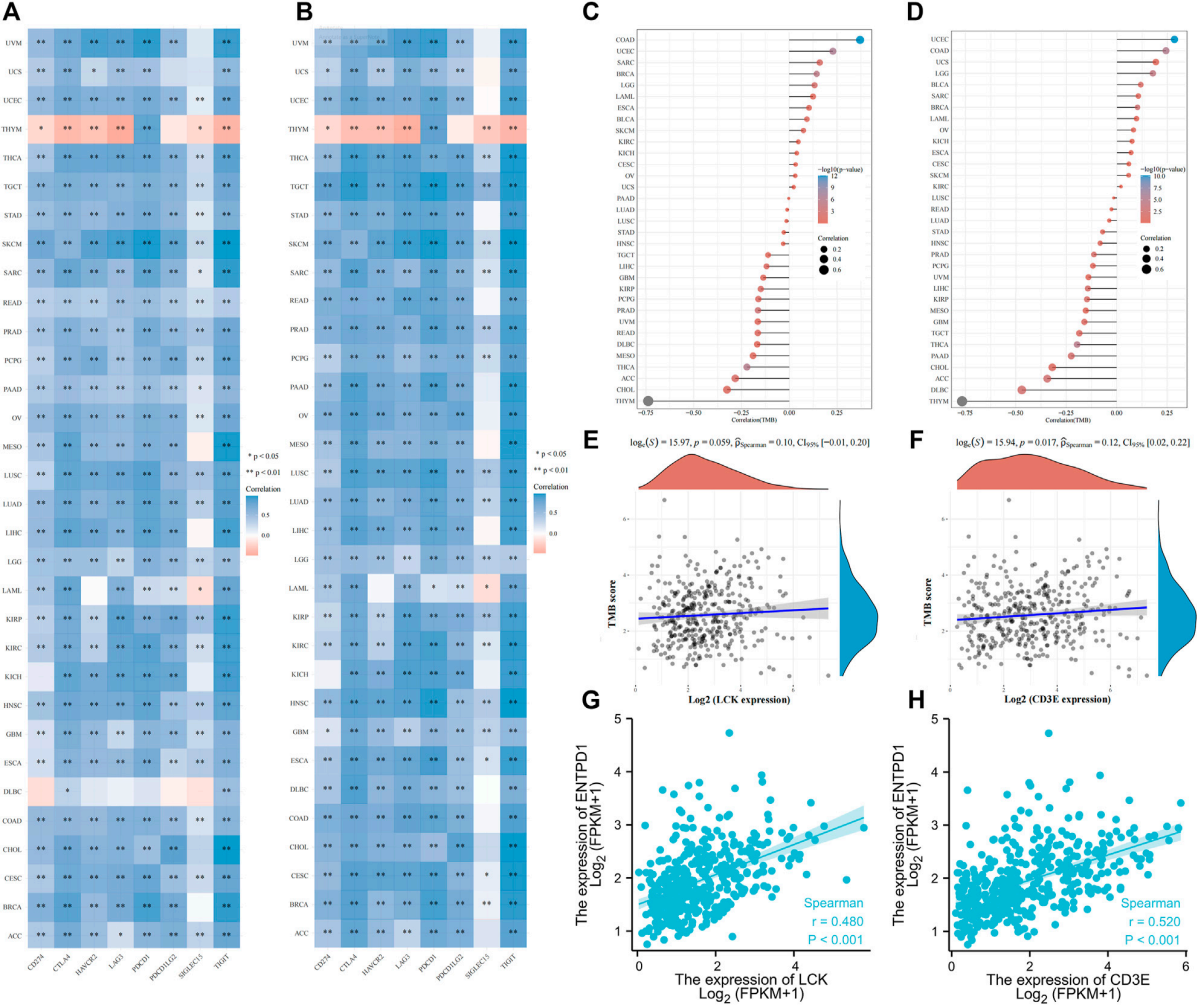
Correlation of LCK/CD3E with immune checkpoint genes and tumor mutation burden across pan-cancers. **(A–B)** Correlation of LCK/CD3E with immune checkpoint genes across pan-cancers. **(C–D)** Correlation of LCK/CD3E with tumor mutation burden across pan-cancers. **(E–F)** Spearman correlation between LCK/CD3E and tumor mutation burden in bladder cancer. **(G–H)** Spearman correlation between ENTPD1 (CD39) with LCK/CD3E in bladder cancer.

Interestingly, LCK and CD3E had a generally positive correlation with those eight immune checkpoint genes for pan-cancers, except for thymoma (Figures 7A, B). Pan-cancer analysis also revealed a positive correlation between LCK/CD3E and TMB in several types of cancers, despite the coefficients being small (Figures 7C, D). Further analyses showed that the correlation of LCK and CD3E with TMB in bladder cancer was weak, although a statistical significance was reached (Figures 7E, F). Last but not least, there was a moderate correlation between CD39, a previously reported gene predictive of response rate to PD-1 inhibitors, with LCK (r = 0.48, *p* < 0.001) and CD3E (r = 0.52, *p* < 0.001) (Figures 7G, H).

### Prognostic Value of LCK and CD3E for MIBC in Predicting Immune Response and Survival After Immunotherapy Among Two Independent Validation Cohorts

**FIGURE 8:**
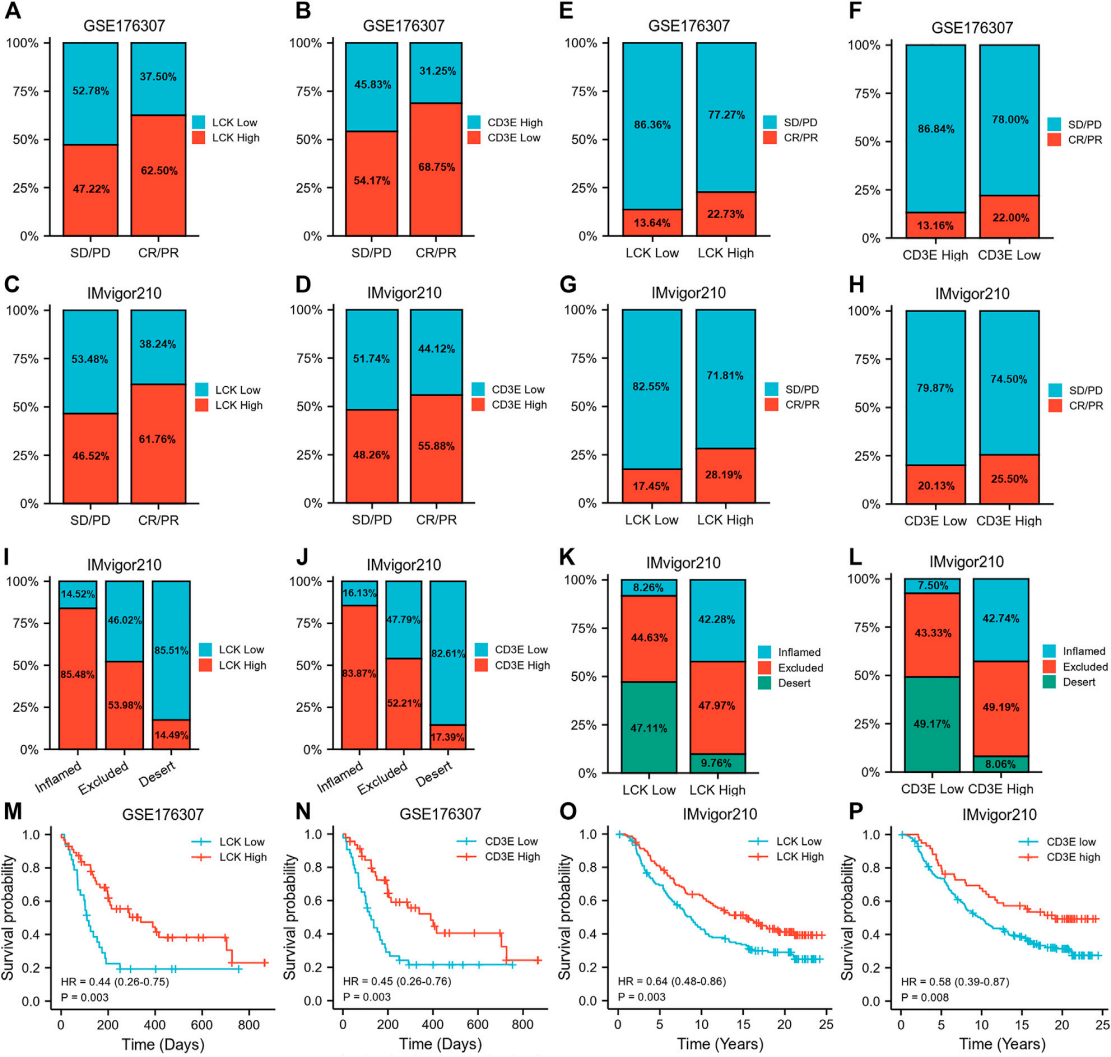
Prognostic value of LCK and CD3E for MIBC in predicting immune response and survival after immunotherapy among two independent validation cohorts. **(A–D)** Percentage of high/low LCK/CD3E expression in responders and non-responders. **(E–H)** Proportion of responder and non-responder in high/low-LCK/CD3E groups. **(I–J)** Percentage of high/low-LCK/CD3E expression in different immune phenotypes. **(K–L)** Proportion of different immune phenotypes in high/low-LCK/CD3E groups. **(M–P)** Kaplan–Meier curves showing the prognostic value of LCK and CD3E for MIBC overall survival in GSE176306 and IMvior210.

Figures 8A, B show a greater percentage of high LCK (62.50 *vs.* 47.22%) and high CD3E (68.75% *vs.* 54.17%) in MIBC patients defined as immunotherapy responders in GSE176307 dataset, which was consistent with the tendency in the IMvigor210 cohort (LCK: 61.76 *vs.* 46.52%, CD3E: 55.88 *vs.* 48.26%, Figures 8C, D). More importantly, the percentage of responders distributed in high-LCK and high-CD3E groups was obviously higher, considering the generally relatively limited response rate of immunotherapy, than the low-expression groups, both in GSE176307 (LCK: 22.73 *vs.* 13.64%, CD3E: 22.00 *vs.* 13.16%, Figures 8E,F) and IMvigor210 cohorts (LCK: 28.19*vs.*vs 17.45%, CD3E: 25.50 *vs.* 20.13%, immune cells), excluded (immune cells accumulated but not efficiently infiltrated) and desert phenotypes (absence of CD8^+^ T cells) (Figures 8I,J). Similarly, high-LCK and high-CD3E group also had more inflamed phenotypes (LCK: 42.28*vs.*vs 8.26%, CD3E: 42.74*vs.*vs 7.50%, Figures 8K, 8L). Supplementary Figure S3 presents a tendency of higher expression of LCK and CD3E in the responder group versus the non-responders (Supplementary Figures S3A–S3D), but statistical significance was not reached. However, a significant trend of LCK/CD3E expression was observed across the immune phenotypes (Supplementary Figure S3E–S3F). Last, the prognostic value of LCK and CD3E was validated by indicating higher expression of LCK (GSE176307: HR 0.44, 95% CI 0.26–0.75, *p* = 0.003; IMvigor210: HR 0.45, 95% CI 0.26–0.76, *p* = 0.003, Figures 8M,N) or CD3E (GSE176307: HR 0.64, 95% CI 0.48–0.86, *p* = 0.003; IMvigor210: HR 0.58, 95% CI 0.39–0.87, *p* = 0.008, Figures 8O,P) and was associated with an improved overall survival for bladder cancer patients receiving immunotherapy.

## Discussion

The current study found the prognostic value of LCK and CD3E for MIBC. A higher expression of LCK/CD3E was indicative of improved survival of MIBC patients. LCK is the major T-cell receptor (TCR) kinase and has selectivity on the four CD3-signaling proteins of TCR (Marth et al., 1985). Early studies demonstrated that LCK was widely expressed in immune cells and was a potential biomarker of malignant tumors (Harr et al., 2010; Till et al., 2017; Zeng et al., 2020). Importantly, through an ionic interaction between basic residue-rich sequences and acidic residues, CD3E is the only CD3 chain that can efficiently interact with LCK (Li et al., 2017). The ionic interaction between LCK and CD3E controls the TCR phosphorylation, which is considered as the initial step in T-cell signaling to trigger adaptive immunity against tumor cells (Li et al., 2017; Hartl et al., 2020). This might also explain our findings of the richer abundance of CD8^+^ T cells in the high-LCK and high-CD3E groups. The CD8^+^ T cell, also known as the cytotoxic T cell, is one of the dominant differentiated T cells. CD8^+^ T cells have been well proved to be the main effector of eliminating tumor cells through the recognition of MHC I molecules binding to the antigen on the surface of tumor cells (Vesely et al., 2011). However, increasing evidence has revealed the shift of CD8^+^ T cells from a functional state to an exhausted state, indicating the high heterogeneity of CD8^+^ T cells (Simoni et al., 2018; Thommen and Schumacher, 2018) and also demanding the combination of CD8 with other biomarkers in predicting prognosis. An early study demonstrated high-affinity neoantigens correlated with better prognosis of hepatocellular carcinoma by activating CD39 ^+^ CD8^+^ T cells (Liu et al., 2020). Therefore, our study assessed the correlation of CD39 with LCK and CD3E, and a positive correlation was found. Altogether, these findings explained, to an extent, the prognostic value of LCK and CD3E for MIBC diagnosis.

Immunotherapy of ICIs has become a common choice for treating advanced cancer worldwide. However, the response rate to ICIs remains low (Doroshow et al., 2021). To provide better guidance of administrating ICIs to patients who are potentially responsive, researchers have longed to explore biomarkers that can predict the benefit of ICI treatment. TMB, the expression level of PD-L1, and the abundance of TICs have been proposed in this context (Goodman et al., 2017; Jia et al., 2018). Nevertheless, controversies still remain in terms of predicting accuracy. For instance, the higher expression of PD-L1 was reported to be predictive of improved survival after ICI immunotherapy, but a small part of patients lacking PD-L1 expression would still benefit from ICIs (Patel and Kurzrock, 2015). The abundance of tumor-infiltrating CD8^+^ T cells also demonstrated to mediate response to immunotherapy. However, CD8^+^ T-cell persistence was observed when it was associated with a CD39-negative state, and a higher proportion of CD39 ^+^ CD8^+^ T cells was correlated with an improved response rate to ICIs (Krishna and Lowery, 2020). Therefore, it looks unreasonable for a single biomarker to predict the survival benefits of ICIs and prognosis for MIBC, which necessitates the inter-biomarker correlation analyses and the integration of different biomarkers. In this context, our study did not only reveal a positive correlation of LCK and CD3E with CD8^+^ T-cell abundance and CD39 expression level but also report a higher expression of several typical immune checkpoint–relevant genes such as PD-1, PD-L1, and CTLA4 in the LCK-high and CD3E-high groups. Notably, LCK- and CD3E-related TMBs were low in our study. These findings taken together suggested the potential of LCK and CD3E in predicting the effect of ICI therapy.

To our knowledge, this is the first study reporting the potential prognostic value of LCK and CD3E in MIBC. However, our findings should not be interpreted without limitations. The first limitation is the retrospective nature of our study. Given that two independent public datasets were employed for validation, multicenter samples would make the findings more convincing. Furthermore, samples used in this study were from the core region of the tumor tissue, making us unable to take different parts of the tumor into the analysis. All those limitations imply well-designed prospective research to validate the prognostic value of LCK and CD3E in clinical practice. Moreover, experiments exploring the potential signaling pathway of LCK/CD3E/TCR, CD8^+^ T cells, CD39, and PD-1/PD-L1 are required.

## Conclusion

CD3E and LCK, two tightly correlated genes in T-cell receptor phosphorylation, were found to be potential biomarkers of MIBC prognosis. Importantly, CD3E and LCK were positively correlated with several regular immunotherapy biomarkers such as TIC infiltration (memory B cells, CD8^+^ T cells, CD4^+^ memory activated, and macrophage M1) and the expression of PD-1, PD-L1, CTLA4, and CD39, which was supported by real-world data from two independent MIBC immunotherapy cohorts.

## Data Availability

The datasets presented in this study can be found in online repositories. The names of the repository/repositories and accession number(s) can be found in the article/Supplementary Material.

## Glossary

ACC: adrenocortical carcinoma
BC: bladder cancer
BLCA: bladder urothelial carcinoma
BRCA: breast invasive carcinoma
CESC: cervical squamous cell carcinoma and endocervical adenocarcinoma
CHOL: cholangiocarcinoma
COAD: colon adenocarcinoma
DEGs: differential expression genes
DLBC: lymphoid neoplasm diffuse large B-cell lymphoma
ESCA: esophageal carcinoma
GBM: glioblastoma multiforme
GEO: Gene Expression Omnibus
GO: gene ontology
HNSC: head and neck squamous cell carcinoma
ICIs: immune checkpoint inhibitors
KEGG: Kyoto Encyclopedia of Genes and Genomes
KICH: kidney chromophobe
KIRC: kidney renal clear cell carcinoma
KIRP: kidney renal papillary cell carcinoma
LAML: acute myeloid leukemia
LGG: brain lower grade glioma
LIHC: liver hepatocellular carcinoma
LUAD: lung adenocarcinoma
LUSC: lung squamous cell carcinoma
MESO: mesothelioma
MIBC: muscle-invasive bladder cancer
OV: ovarian serous cystadenocarcinoma
PAAD: pancreatic adenocarcinoma
PCPG: pheochromocytoma and paraganglioma
PRAD: prostate adenocarcinoma
PPI: protein–protein interaction
READ: rectum adenocarcinoma
SARC: sarcoma
SKCM: skin cutaneous melanoma
STAD: stomach adenocarcinoma
TCGA: The Cancer Genome Atlas
TCR: T-cell receptor
TGCT: testicular germ cell tumors
THCA: thyroid carcinoma
THYM: thymoma
TICs: tumor-infiltrating immune cells
TMB: tumor mutation burden
UCS: uterine carcinosarcoma
UVM: uveal melanoma.
