# Supplementary figures and images for: LCK and CD3E Orchestrate the Tumor Microenvironment and Promote Immunotherapy Response and Survival of Muscle-Invasive Bladder Cancer Patients
Source: Front Cell Dev Biol. 2021 Dec 24;9:748280. doi: 10.3389/fcell.2021.748280 (PMC8740181; doi:10.3389/fcell.2021.748280)

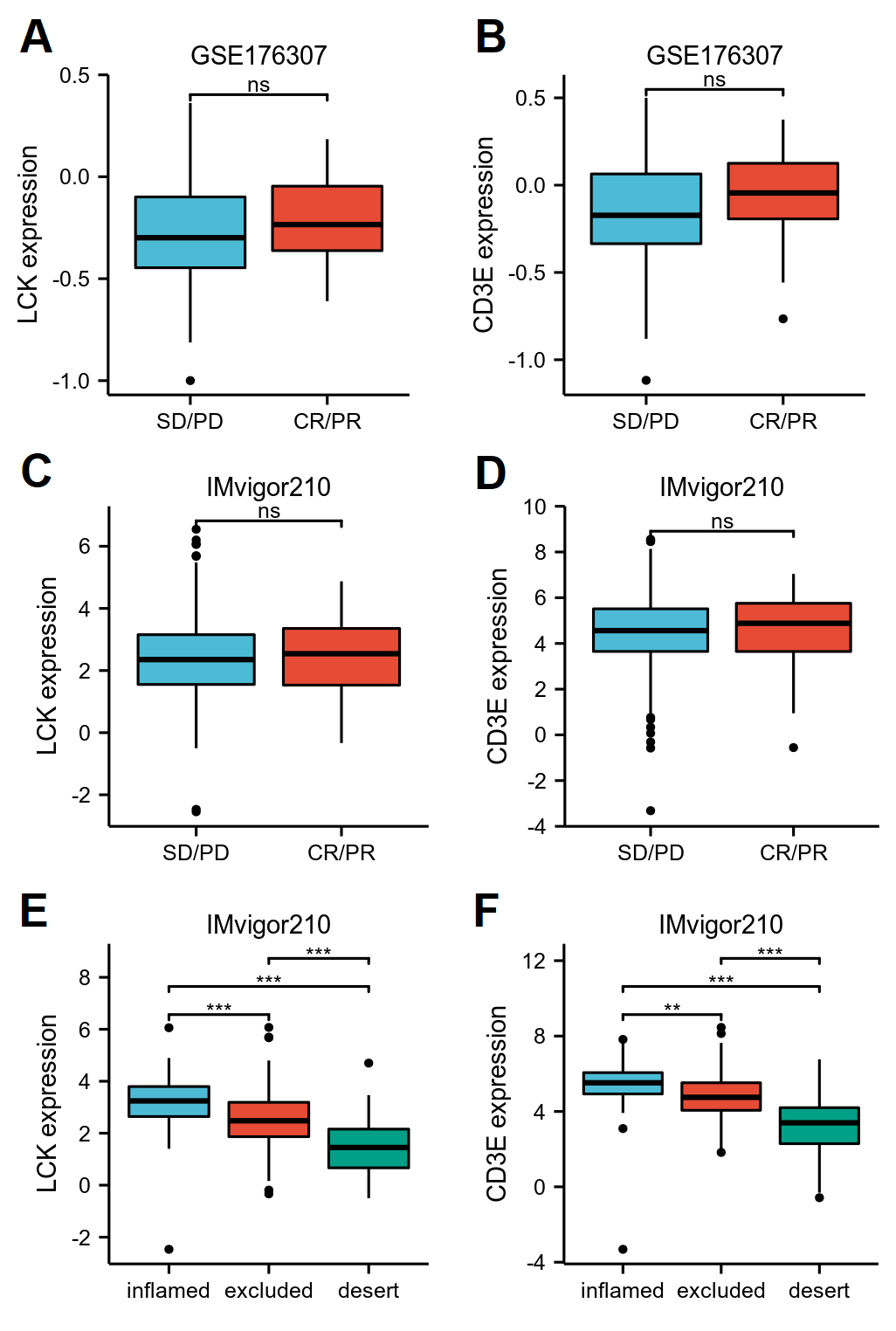

Supplement: Supplementary file 1 [file Image3.TIF]

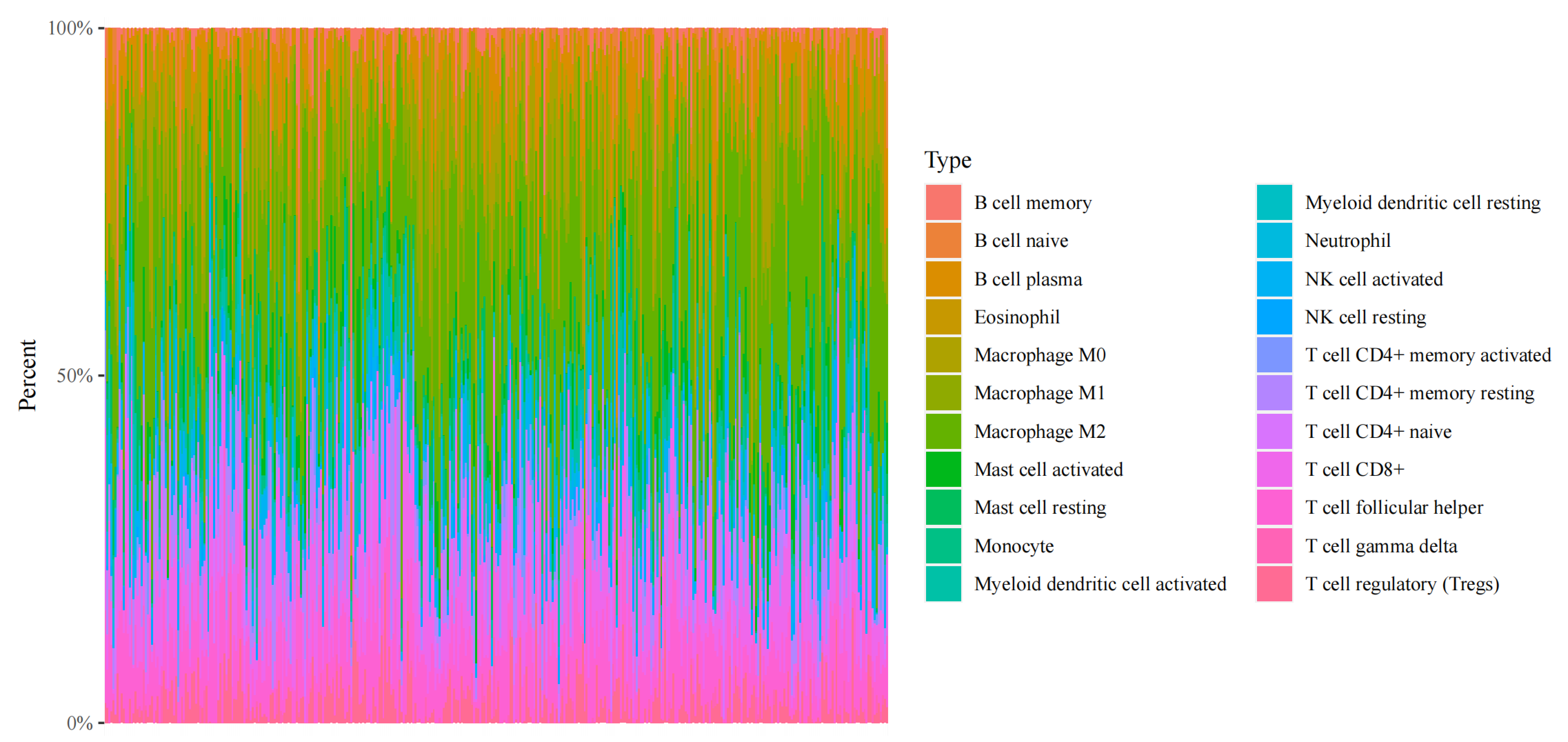

Supplement: Supplementary file 2 [file Image2.TIF]

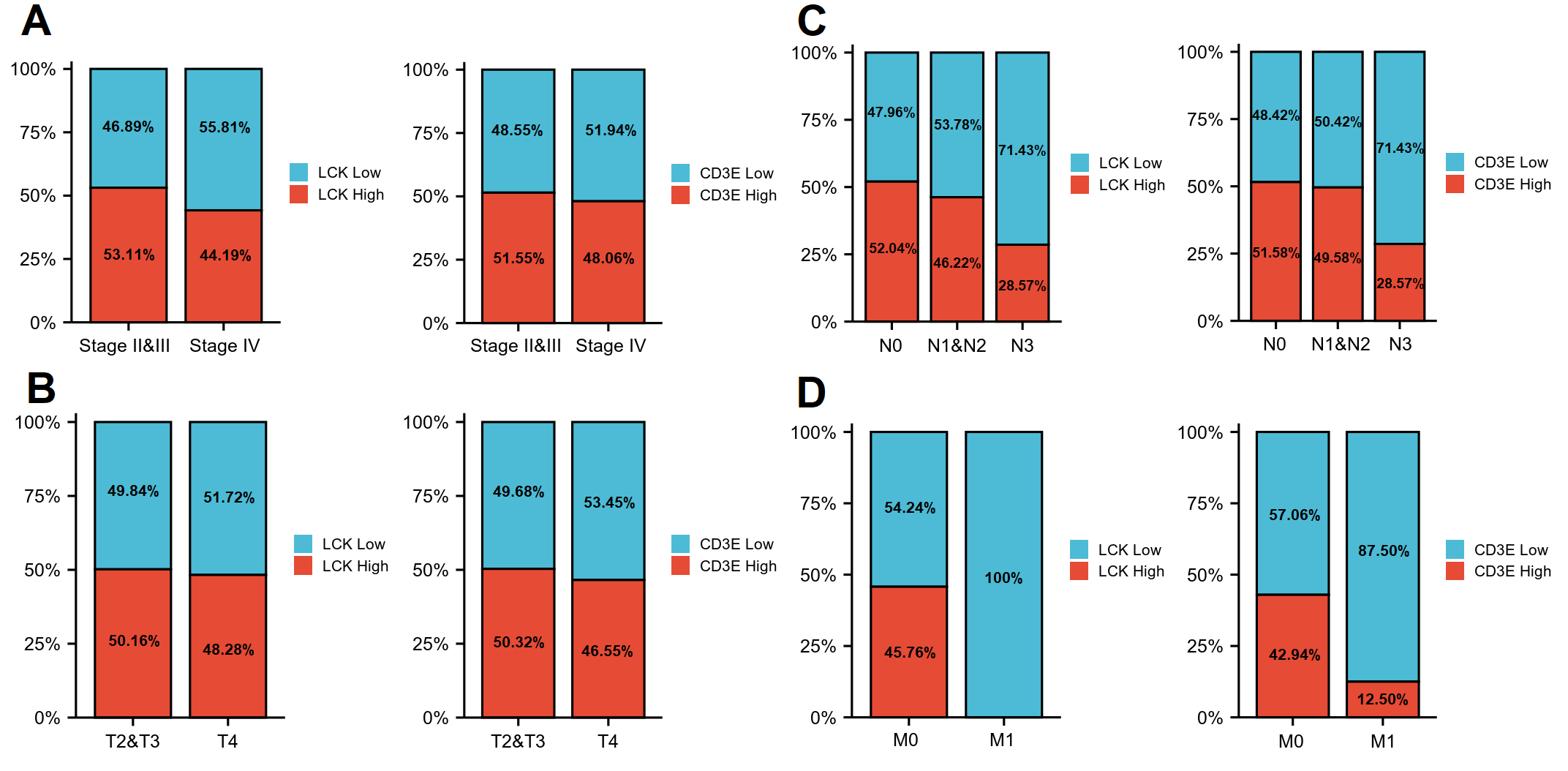

Supplement: Supplementary file 3 [file Image1.TIF]
